# Burden, features and factor associated with disorders of gut–brain axis in Italy: results from the Rome foundation global epidemiology study

**DOI:** 10.1007/s11739-026-04350-w

**Published:** 2026-04-30

**Authors:** Giovanni Marasco, Keren Hod, Luigi Colecchia, Cesare Cremon, Enrico Stefano Corazziari, Ami David Sperber, Olafur Steinn Palsson, Shrikant Ishver Bangdiwala, Giovanni Barbara

**Affiliations:** 1 Department of Medical and Surgical Sciences, University of Bologna, Via Massarenti, 9, 40138 Bologna, Italy; 2 IRCCS Azienda Ospedaliero-Universitaria di Bologna, Bologna, Italy; 3 Department of Nutrition Sciences, Faculty of Health Sciences, Ariel University, Ariel, Israel; 4 Assuta Medical Centers, Tel Aviv, Israel; 5 Centro di Eccellenza di Malattie Gastrointestinali ed Endocrino-Metaboliche, Ospedale Isola Tiberina, Rome, Italy; 6 Faculty of Health Sciences, Ben-Gurion University of the Negev, Beer-Sheva, Israel; 7 Center for Functional GI and Motility Disorders, University of North Carolina-Chapel Hill, Chapel Hill, NC USA; 8 Department of Health Research Methods, Evidence and Impact, McMaster University, Hamilton, ON Canada; 9 Population Health Research Institute, McMaster University, Hamilton, ON Canada

**Keywords:** Disorders of gut–brain interaction, Irritable bowel syndrome, Functional dyspepsia, Italy

## Abstract

Disorders of gut–brain interaction (DGBI) are prevalent and burdensome conditions. This study aimed to assess the prevalence, regional distribution, and associated factors of DGBI in Italy, and compare findings with the rest of Europe. Data were drawn from the Rome Foundation Global Epidemiology Study. A representative sample of 2063 Italian adults completed a internet-based questionnaire assessing DGBI presence, psychological distress, somatic symptoms, dietary habits, and healthcare utilization. Comparative analysis was conducted within the country and with other European countries. Multivariate logistic regression identified independent associated factors in Italy. Overall, 44.2% of Italian participants met criteria for at least one DGBI with higher prevalence in women and individuals aged 18–39 years. The South and Islands showed the highest DGBI prevalence. Functional constipation (13.9%) and functional dyspepsia (9.1%) were the most common DGBI, while irritable bowel syndrome affected 5.0% of the population. Psychological distress, somatic symptom burden, female sex, and younger age were independently associated with DGBI, while living in small rural communities showed an inverse association. Compared to the rest of Europe, Italy showed higher DGBI rates across multiple gastrointestinal regions and more frequent overlapping DGBI. DGBI represent a substantial burden in Italy compared with the rest of Europe, with marked regional and psychosocial gradients.

**Supplementary Information:**

The online version contains supplementary material available at 10.1007/s11739-026-04350-w.

## Introduction

The disorders of gut–brain interaction (DGBI) are defined by a range of gastrointestinal symptoms caused by any combination of motility disturbance, visceral hypersensitivity, modified mucosal and immunological function, altered gut microbiota, altered central nervous system processing and genetic polymorphism. [1] Irritable bowel syndrome (IBS), functional dyspepsia (FD), functional constipation (FC), and functional diarrhea (FDr) are the most common conditions that fall within the DGBI spectrum. [1] These disorders affect up to 30–40% of the general population in Western countries, and their prevalence decreases with age. [2, 3] Although the burden of individual DGBI in Italy such as IBS [4] or FD [5] has been quantified previously, no study to date has provided a thorough, country‐specific assessment of the burden of DGBI. The Rome Foundation Global Epidemiology Study (RFGES) evaluating a total of 22 DGBI revealed that 40.3% out of the 54,127 participants in 26 countries, including Italy, met diagnostic criteria for at least one DGBI, suggesting that these diseases represent a major global burden of these disorders [6]. The RFGES study in Italy included 2063 respondents, of whom about 40% fulfilled the criteria for at least one DGBI. [6] In the context of European countries, Italy, and other southern European countries around the Mediterranean Sea are renowned throughout the world for their Mediterranean lifestyle, which has been associated with general well-being and reduced rates of psychological disorders, even though the latter were previously associated with higher prevalence rates of DGBI. [7] Within southern European countries, however, there are regional differences in the extent of adherence to the Mediterranean diet, lifestyle, and overall psychosocial well-being. [6]

Within this study, we aimed first to report the burden of DGBI in Italy, stratified by regions, age, gender, and living area. Then, we aimed to identify differences in the burden of DGBI between Italy and the rest of Europe. Finally, we compared DGBI risk factors among Italian and other European populations, focusing on the most reported DGBI, including FD, IBS, FC, and FDr.

## Methods

### Study design and participants

This study was based on data from the RFGES, a large multi-national cross-sectional survey aimed at assessing the prevalence and burden of DGBI. The methodology of the RFGES has been published previously and described in detail elsewhere. [6] In brief, this study collected data from 54,127 adult participants (≥ 18 years) across 26 countries via either internet-based or face-to-face interviews. For the current analysis, only data obtained through internet-based self-administered questionnaires were used. Participants were drawn from a pre-existing online panel of individuals who had consented to participate in health-related research. To minimize selection bias, the survey was introduced as a general health study without any mention of gastrointestinal or psychological symptoms. All participants provided electronic informed consent prior to participation, and data were collected anonymously. The study received ethics approval or was deemed exempt in each participating country, including Italy.

In Italy, a representative sample of 2,063 adults was recruited. To enable region-specific analyses and account for potential geographic variability, the Italian sample was further categorized into four main macro-regions based on administrative and geographic divisions: Northwest (Liguria, Lombardy, Piedmont, Aosta Valley), Northeast (Emilia-Romagna, Friuli-Venezia Giulia, Trentino-Alto Adige, Veneto), Center (Lazio, Marche, Tuscany, Umbria), and South and Islands (Abruzzo, Basilicata, Calabria, Campania, Molise, Apulia, Sardinia and Sicily). To provide contextual insights, comparisons were subsequently made with participants from other European countries.

### Data collection and questionnaires

DGBI were defined as the presence of at least one DGBI, diagnosed using validated modules from the Rome IV Diagnostic Questionnaire [8] (alongside a checklist to rule out organic diseases and surgeries that could account for gastrointestinal symptoms. DGBI were categorized by anatomical region (esophageal, gastroduodenal, bowel, anorectal), and specific diagnoses, including IBS and its subtypes [IBS with predominant constipation (IBS-C), IBS with predominant diarrhea (IBS-D), mixed IBS (IBS-M), and undefined IBS (IBS-U)], FD, FC, and FDr, were assessed individually. A detailed description of the administered questionnaires and classifications used in text are available in the supplementary materials. In brief, somatic symptoms were evaluated using the Patients Health Questionnaire-12 (PHQ-12) [9], physical and mental health-related quality of life was assessed via the Patient-Reported Outcomes Measurement Information System (PROMIS) Global-10 [10], the work productivity and activity Impairment was assessed via the General Health (WPAI:GH) questionnaire [11], psychological distress was evaluated using the Patients Health Questionnaire-4 (PHQ-4) [12], the weekly consumption frequency of various food groups was assessed via the Food Frequency Questionnaire (FFQ).

### Statistical analysis

All eligible participants from internet interviews were included. Data normality was evaluated using the Kolmogorov–Smirnov test. Normality assessments were supplemented by visual inspection of histograms and Q–Q plots. Normally distributed continuous variables were reported as mean and 95% confidence interval (CI), otherwise, as median and 95% CI. Categorical variables were reported as percentages and 95% CI. Age categories were identified as follows: 18–39 years vs. 40–64 vs 65 + . The prevalence rate of each one of the 22 DGBI was calculated for the total Italian population and was stratified by regions, age, gender, and living area. Differences between European and Italian DGBI patients were calculated using Student's t test, Mann–Whitney, Fisher's test, and chi-square as appropriate.

Multiple multivariable logistic regression models using the forward stepwise selection method were applied to clarify the role of demographics, clinical and psychological features, and dietary factors for at least one DGBI, FD, or IBS occurrence in Italy. The estimated odds ratios (OR) with their 95% CI were calculated. Independent variables that were inserted into these models were those with a significant association with DGBI (*p* < 0.1) as found in a previous univariate analysis. Independent variables that were highly correlated (r ≥ 0.600) were not included in these multivariable logistic regression models simultaneously, even if they were significantly associated with DGBI, to avoid multicollinearity. Associations with a p-value < 0.05 were considered statistically significant. Statistical analysis was performed using STATA17 (Stata Corp., College Station, TX, USA) and the SPSS Statistics for Windows package, version 29.0 (IBM Corp. Released 2019, Armonk, NY).

## Results

### Prevalence of DGBI in Italy

**Fig. 1:**
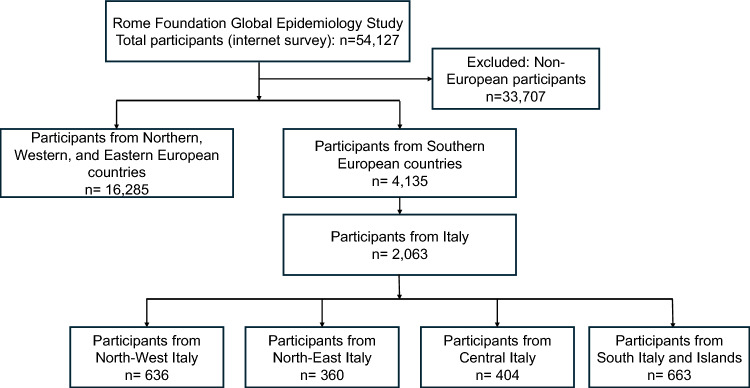
Flow chart showing the selection of participants for the analysis from the Rome Foundation Global Epidemiology Study (*N* = 54,127). Participants from countries outside of Europe (*n* = 33,707) were excluded. The remaining participants (*n* = 20,420) were grouped into: (1) Northern (Sweden, the United Kingdom), Western (Belgium, France, Germany, the Netherlands), and Eastern European countries (Poland, Romania); and (2) Southern European countries (Italy and Spain). Italian participants were further classified by macro-regions based on geographic divisions: Northwest Italy (*n* = 636, includes Liguria, Lombardy, Piedmont, and Aosta Valley), Northeast Italy (*n* = 360, includes Emilia-Romagna, Friuli-Venezia Giulia, Trentino-Alto Adige, and Veneto), Central Italy (*n* = 404, includes Lazio, Marche, Tuscany, and Umbria), and South Italy and Islands (*n* = 663, includes Abruzzo, Basilicata, Calabria, Campania, Molise, Apulia, Sicily, and Sardinia)

Of the total 54,127 participants included in the RFGES, 20,420 participants represented European countries and were stratified into groups: 16,285 participants from northern, western, and eastern European countries; 4,135 from southern European countries; and, within the southern European sample, 2,063 participants were specifically from Italy. The Italian participants were further classified into four subgroups: Northwest Italy (*n* = 636), Northeast Italy (*n* = 360), the central region of Italy (*n* = 404), and South Italy and Islands (*n* = 663) (Fig. 1).

**Fig. 2:**
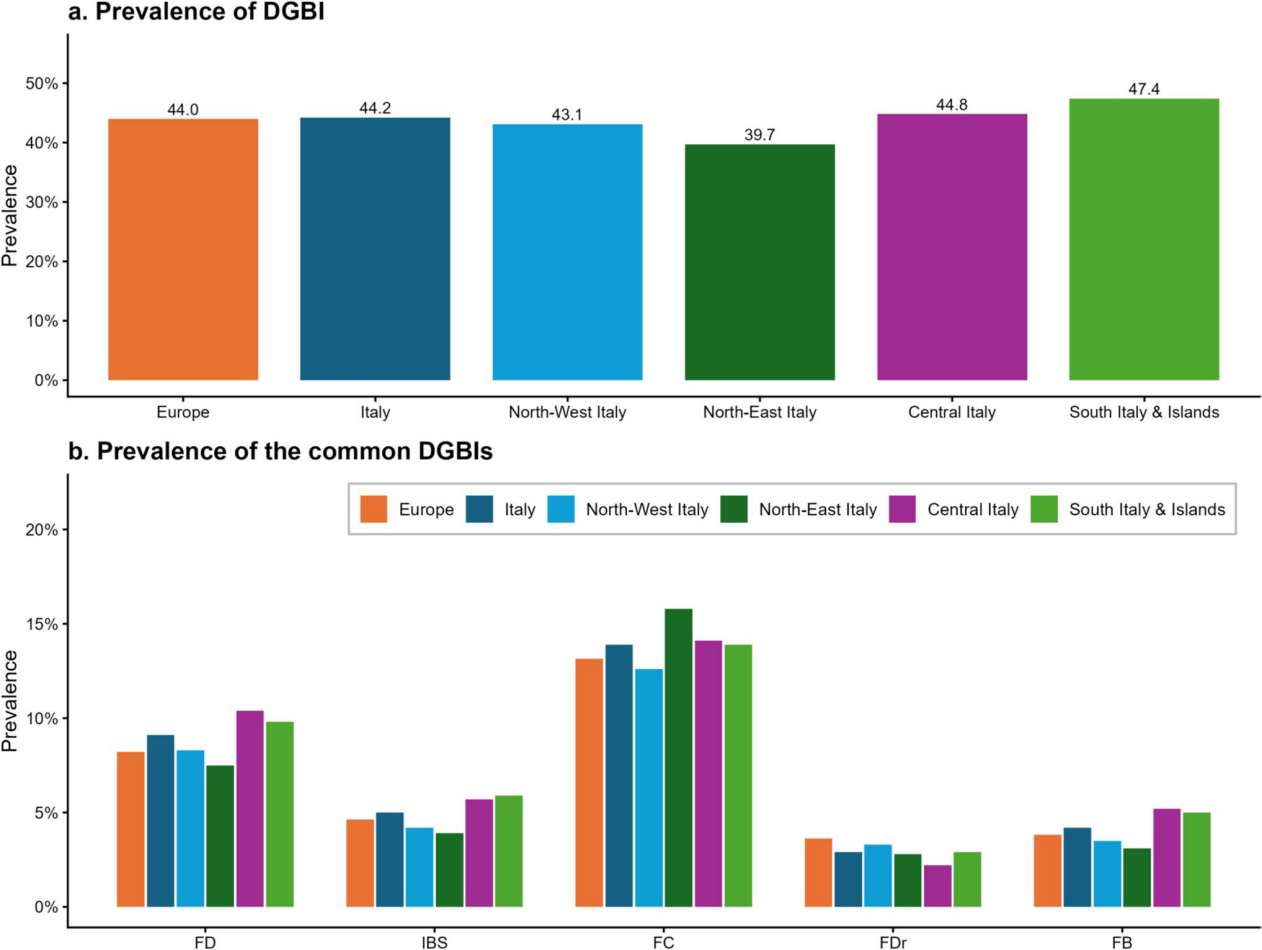
**A** Prevalence of reporting at least one DGBI in Europe, Italy and Italian macro-regions. **B** Prevalence of the most common specific DGBI in Europe, Italy and Italian macro-regions. Abbreviations: FD, functional dyspepsia; IBS, irritable bowel syndrome; FDr, functional diarrhea; FC, functional constipation; FB, functional bloating

Of the total Italian population, 44.2% had at least one DGBI (Fig. 2A). This prevalence was significantly higher in women (53.8%) compared to men (34.7%) (*p* < 0.001). Age also played a crucial role, with the highest prevalence observed in the 18–39 age group (51.6%), followed by the 40–64 age group (43.2%), and the 65 + age group (31.3%) (*p* < 0.001). Additionally, the prevalence of DGBI was higher in urban areas (44.8%) compared to rural areas (39.0%) although the difference was not statistically significant (Table 1).

**Table 1:** Prevalence rate (%, 95% CI) for 22 DGBI and their overlaps among the Italian population by gender, age, and living area

	Total population (2063)	Gender			Age group (years)				Living area		
		Male (*n* = 1035)	Female (*n* = 1028)	*P* value	18–39 (*n* = 826)	40–64 (*n* = 828)	65 + (*n* = 409)	*P* value	Urban^1^ (*n* = 1868)	Rural^2^ (*n* = 195)	*P* value
Any DGBI	44.2 (42.0–46.4)	34.7 (31.8–37.7)***	53.8 (50.7–56.9)***	< 0.001	51.6 (48.1–55.0)***	43.2 (39.8–46.7)***	31.3 (26.8–36.0)***	< 0.001	44.8 (42.5–47.0)	39.0 (32.1–46.2)	0.130
Any esophageal DGBI	8.4 (7.2–9.7)	6.6 (5.1–8.3)**	10.2 (8.4–12.2)**	0.003	9.0 (7.1–11.1)*	9.5 (7.6–11.7)*	4.9 (3.0–7.5)*	0.016	8.6 (7.3–9.9)	6.7 (3.6–11.1)	0.417
Functional chest pain	1.6 (1.1–2.2)	0.9 (0.4–1.6)**	2.3 (1.5–3.5)**	0.008	1.7 (0.9–2.8)	1.8 (1.0–3.0)	1.0 (0.3–2.5)	0.525	1.6 (1.1–2.3)	1.5 (0.3–4.4)	> 0.999
Functional heartburn	1.7 (1.2–2.4)	1.9 (1.2–3.0)	1.5 (0.8–2.4)	0.496	1.9 (1.1–3.1)	2.1 (1.2–3.3)	0.5 (0.1–1.8)	0.106	1.8 (1.2–2.5)	1.0 (0.1–3.7)	0.768
Reflux hypersensitivity	1.5 (1.0–2.1)	1.3 (0.7–2.1)	1.8 (1.0–2.8)	0.372	1.8 (1.0–3.0)	1.6 (0.8–2.7)	0.7 (0.2–2.1)	0.332	1.4 (1.0–2.1)	2.1 (0.6–5.2)	0.529
Globus	1.6 (1.1–2.3)	1.0 (0.5–1.8)*	2.3 (1.5–3.5)*	0.015	1.8 (1.0–3.0)	1.7 (0.9–2.8)	1.2 (0.4–2.8)	0.737	1.7 (1.1–2.3)	1.5 (0.3–4.4)	> 0.999
Functional dysphagia	4.2 (3.3–5.1)	3.4 (2.4–4.7)	5.0 (3.7–6.5)	0.078	4.6 (3.3–6.3)	4.6 (3.3–6.2)	2.4 (1.2–4.5)	0.150	4.2 (3.3–5.2)	4.1 (1.8–7.9)	> 0.999
Any gastroduodenal DGBI	12.7 (11.3–14.3)	10.0 (8.2–11.9)***	15.6 (13.4–17.9)***	< 0.001	15.9 (13.4–18.5)***	12.4 (10.3–14.9)***	7.1 (4.8–10.0)***	< 0.001	12.8 (11.4–14.5)	11.8 (7.6–17.2)	0.736
Functional dyspepsia	9.1 (7.9–10.4)	7.1 (5.6–8.8)**	11.1 (9.2–13.2)**	0.002	11.5 (9.4–13.9)***	8.9 (7.1–11.1)***	4.4 (2.6–6.9)***	< 0.001	9.0 (77.7–10.4)	9.7 (6.0–14.8)	0.695
PDS	8.1 (7.0–9.4)	6.3 (4.9–7.9)**	9.9 (8.2–11.9)**	0.003	10.0 (8.1–12.3)**	8.0 (6.2–10.0)**	4.4 (2.6–6.9)**	0.003	8.0 (6.8–9.4)	8.7 (5.2–13.6)	0.681
EPS	3.2 (2.5–4.1)	2.6 (1.7–3.8)	3.9 (2.8–5.3)	0.107	4.2 (3.0–5.8)**	3.6 (2.5–5.1)**	0.5 (0.1–1.8)**	0.002	3.3 (2.6–4.2)	2.6 (0.8–5.9)	0.831
Belching disorder	1.1 (0.7–1.7)	1.3 (0.7–2.1)	1.0 (0.5–1.8)	0.676	1.2 (0.6–2.2)	0.8 (0.3–1.7)	1.5 (0.5–3.2)	0.584	1.2 (0.8–1.8)	0.0 (0.0–1.9)	0.160
Rumination syndrome	3.8 (3.0–4.7)	2.5 (1.6–3.7)**	5.1 (3.8–6.6)**	0.003	3.3 (2.2–4.7)	4.5 (3.2–6.1)	3.4 (1.9–5.7)	0.403	3.9 (3.1–4.9)	2.6 (0.8–5.9)	0.433
Chronic nausea/vomiting	1.0 (0.6–1.6)	0.8 (0.3–1.5)	1.3 (0.7–2.2)	0.282	1.5 (0.8–2.5)	0.8 (0.3–1.7)	0.5 (0.1–1.8)	0.231	1.1 (0.7–1.6)	0.5 (0.0–2.8)	0.714
Cyclic vomiting syndrome	1.8 (1.3–2.5)	1.2 (0.6–2.0)*	2.4 (1.6–3.6)*	0.031	2.8 (1.8–4.1)**	1.6 (0.8–2.7)**	0.2 (0.0–1.4)**	0.005	1.7 (1.1–2.3)	3.1 (1.1–6.6)	0.155
Cannabinoid hyperemesis	0.0 (0.0–0.2)	0.0 (0.0–0.4)	0.0 (0.0–0.4)	> 0.999	0.0 (0.0–0.4)	0.0 (0.0–0.4)	0.0 (0.0–0.9)	> 0.999	0.0 (0.0–0.2)	0.0 (0.0–1.9)	> 0.999
Any bowel DGBI	37.2 (35.1–39.3)	28.1 (25.4–31.0)***	46.3 (43.2–49.9)***	< 0.001	44.3 (40.9–47.8)***	36.6 (33.3–40.0)***	24.0 (19.9–28.4)***	< 0.001	37.5 (35.3–39.8)	33.8 (27.2–41.0)	0.350
Rome IV IBS	5.0 (4.1–6.0)	3.9 (2.8–5.2)*	6.1 (4.7–7.8)*	0.020	6.7 (5.1–8.6)***	5.0 (3.6–6.7)***	1.7 (0.7–3.5)***	< 0.001	5.2 (4.3–6.4)	2.6 (0.8–5.9)	0.119
IBS-C	1.5 (1.0–2.1)	1.2 (0.6–2.0)	1.8 (1.0–2.8)	0.276	1.9 (1.1–3.1)	1.6 (0.8–2.7)	0.2 (0.0–1.4)	0.061	1.6 (1.0–2.2)	0.5 (0.0–2.8)	0.355
IBS-D	2.2 (1.6–2.9)	1.8 (1.1–2.9)	2.5 (1.7–3.7)	0.295	3.0 (2.0–4.4)	1.8 (1.0–3.0)	1.2 (0.4–2.8)	0.080	2.2 (1.6–3.0)	1.5 (0.3–4.4)	0.795
IBS-M	1.2 (0.8–1.8)	0.7 (0.3–1.4)*	1.8 (1.0–2.8)*	0.028	1.6 (0.8–2.7)	1.3 (0.7–2.4)	0.2 (0.0–1.4)	0.123	1.3 (0.8–1.9)	0.5 (0.0–2.8)	0.506
IBS-U	0.1 (0.0–0.4)	0.2 (0.0–0.7)	0.1 (0.0–0.5)	> 0.999	0.1 (0.0–0.7)	0.2 (0.0–0.9)	0.0 (0.0–0.9)	0.561	0.2 (0.0–0.5)	0.0 (0.0–1.9)	> 0.999
Functional constipation	13.9 (12.4–15.4)	8.1 (6.5–9.9)***	19.6 (17.3–22.2)***	< 0.001	15.9 (13.4–18.5)*	13.9 (11.6–16.4)*	9.8 (7.1–13.1)*	0.014	13.7 (12.1–15.3)	15.9 (11.1–21.8)	0.384
Opioid-induced constipation	1.1 (0.7–1.7)	1.0 (0.5–1.8)	1.3 (0.7–2.2)	0.538	0.6 (0.2–1.4)	1.4 (0.8–2.5)	1.5 (0.5–3.2)	0.197	1.1 (0.7–1.7)	1.0 (0.1–3.7)	> 0.999
Functional diarrhea	2.9 (2.2–3.7)	3.0 (2.0–4.2)	2.7 (1.8–3.9)	0.792	3.5 (2.4–5.0)*	3.1 (2.1–4.6)*	1.0 (0.3–2.5)*	0.035	2.8 (2.1–3.6)	3.6 (1.5–7.3)	0.497
Functional abdominal bloating/distention	4.2 (3.4–5.2)	2.6 (1.7–3.8)***	5.8 (4.5–7.4)***	< 0.001	4.8 (3.5–6.5)	4.6 (3.3–6.2)	2.2 (1.0–4.1)	0.074	4.4 (3.6–5.5)	2.1 (0.6–5.2)	0.134
Unspecified functional bowel disorder	10.1 (8.9–11.5)	9.6 (7.8–11.5)	10.7 (8.9–12.8)	0.422	12.3 (10.2–14.8)*	8.9 (7.1–11.1)*	8.1 (5.6–11.1)*	0.022	10.3 (9.0–11.8)	8.2 (4.8–13.0)	0.385
Centrally mediated abdominal pain syndrome	0.0 (0.0–0.3)	0.0 (0.0–0.4)	0.1 (0.0–0.5)	0.498	0.0 (0.0–0.4)	0.1 (0.0–0.7)	0.0 (0.0–0.9)	0.474	0.0 (0.0–0.3)	0.1 (0.0–1.9)	> 0.999
Functional biliary pain	0.1 (0.0–0.3)	0.0 (0.0–0.4)	0.2 (0.0–0.7)	0.248	0.2 (0.0–0.9)	0.0 (0.0–0.4)	0.0 (0.0–0.9)	0.223	0.1 (0.0–0.4)	0.0 (0.0–1.9)	> 0.999
Any anorectal DGBI	10.4 (9.1–11.8)	7.9 (6.4–9.7)***	12.9 (10.9–15.1)***	< 0.001	11.0 (0.9–13.4)	10.4 (8.4–12.7)	9.3 (6.7–12.5)	0.646	10.6 (9.2–12.1)	8.7 (5.2–13.6)	0.462
Fecal incontinence	0.8 (0.4–1.3)	0.7 (0.3–1.4)	0.9 (0.4–1.7)	0.627	0.5 (0.1–1.2)	0.8 (0.3–1.7)	1.2 (0.4–2.8)	0.363	0.8 (0.5–1.3)	0.5 (0.0–2.8)	> 0.999
Levator ani syndrome	1.6 (1.1–2.2)	1.0 (0.5–1.8)*	2.2 (1.4–3.3)*	0.023	1.2 (0.6–2.2)	1.8 (1.0–3.0)	2.0 (0.8–3.8)	0.507	1.6 (1.1–2.3)	1.5 (0.3–4.4)	> 0.999
Proctalgia fugax	8.2 (7.0–9.5)	6.5 (5.1–8.1)**	9.9 (8.2–11.9)**	0.005	9.3 (7.4–11.5)	8.0 (6.2–10.0)	6.4 (4.2–9.2)	0.193	8.4 (7.1–9.7)	6.7 (3.6–11.1)	0.493
Number of DGBI/person, mean (95% CI)	0.76 (0.71–0.80)	0.57 (0.51–0.64)***	0.94 (0.86–1.01)***	< 0.001	0.87 (0.79–0.96)***	0.76 (0.68–0.84)***	0.49 (0.41–0.58)***	< 0.001	0.76 (0.71–0.81)	0.69 (0.53–0.85)	0.189
Number of GI anatomic regions with DGBI^3^, mean (95% CI)	0.68 (0.65–0.73)	0.52 (0.47–0.58)***	0.85 (0.79–0.91)***	< 0.001	0.80 (0.73–0.87)***	0.68 (0.62–0.75)***	0.45 (0.38–0.53)***	< 0.001	0.69 (0.65–0.74)	0.61 (0.48–0.74)	0.180
Number of GI anatomic regions with DGBI^3^											
One GI anatomic regions with DGBI	27.0 (25.1–29.0)	21.9 (19.4–24.6)***	32.1 (29.3–35.1)***	< 0.001	32.0 (28.8–35.3)***	25.4 (22.4–28.5)***	20.3 (16.5–24.5)***	< 0.001	27.5 (25.5–29.6)	22.1 (16.4–28.5)	0.108
Two GI anatomic regions with DGBI	11.4 (10.1–12.8)	8.7 (7.1–10.6)***	14.1 (12.0–16.4)***	< 0.001	12.8 (10.6–15.)	11.4 (9.3–13.7)	8.6 (6.0–11.7)	0.084	11.2 (9.8–12.8)	12.8 (8.5–18.3)	0.479
Three GI anatomic regions with DGBI	4.3 (3.5–5.3)	3.0 (2.0–4.2)**	5.6 (4.3–7.2)**	0.003	4.6 (3.3–6.3)*	5.2 (3.8–6.9)*	2.0 (0.8–3.8)***	0.027	4.4 (3.6–5.5)	3.1 (1.1–6.6)	0.461
Four GI anatomic regions with DGBI	1.5 (1.0–2.1)	1.1 (0.5–1.9)	1.9 (1.2–3.0)	0.106	2.2 (1.3–3.4)***	1.3 (0.7–2.4)***	0.5 (0.1–1.8)***	0.062	1.6 (1.0–2.2)	1.0 (0.1–3.7)	0.763

^1^Urban area comprises > 2500 residents

^2^Rural area comprises < 2500 residents

^3^One patient can have multiple DGBI in the same region; GI anatomic regions are: esophageal, gastroduodenal, bowel, and anorectal

Abbreviations: CI, Confidence interval; DGBI, Disorder of the brain-gut interaction; EPS, Epigastric pain syndrome; IBS-C, Constipation predominant irritable bowel syndrome; IBS-D, Diarrhea predominant irritable bowel syndrome; IBS-M, Mixed irritable bowel syndrome; IBS-U, Unspecified irritable bowel syndrome;; PDS, Postprandial distress syndrome

^*^Comparisons within Italian population, *P* value < 0.05; ** Comparisons within Italian population, *P* value < 0.01; *** Comparisons within Italian population, *P* value < 0.001

All results are presented as percentages with 95% confidence intervals (95% CI), unless otherwise stated

The prevalence of DGBI varied across different macro-regions in Italy. The highest prevalence was found in the South and Islands (47.4%), followed by the Central region (44.8%), Northwest (43.1%), and Northeast (39.7%)(Fig. 2A). Considering DGBI of specific anatomical regions of the gastrointestinal tract, their prevalence did not significantly differ between the Italian macro-regions except for esophageal DGBI that were significantly higher in the Central region (10.6%), followed by the South and Islands (10.1%), the Northwest (6.9%) and the Northeast (5.3%) (*p* < 0.01) (Table 2a). While the proportion of individuals with DGBI limited to a single gastrointestinal region was broadly comparable across regions (25.3–28.7%), the prevalence of DGBI overlap showed marked geographic variation. In particular, the South and Islands exhibited the highest proportion of individuals with DGBI involving two (12.8%), three (5.1%), or four gastrointestinal regions (2.0%). In contrast, Northwest and Northeast Italy consistently showed lower proportions of individuals with DGBI, affecting three or more gastrointestinal regions, indicating a lower burden of overlapping DGBI in northern regions.

**Table 2:** Prevalence rate (%, 95% CI) for 22 DGBI among the Italian population by region

	Northwest (*n* = 636)	Northeast (*n* = 360)	Center (n = 404)	South and Islands (*n* = 663)	*P* value
Any DGBI	43.1 (39.2–47.0)	39.7 (34.6–45.0)	44.8 (39.9–49.8)	47.4 (43.5–51.2)	0.112
Any esophageal DGBI	6.9 (5.1–9.2)**	5.3 (3.2–8.1)**	10.6 (7.8–14.1)**	10.1 (7.9–12.7)**	0.009
Functional chest pain	0.9 (0.3–2.0)	1.1 (0.3–2.8)	1.7 (0.7–3.5)	2.4 (1.4–3.9)	0.163
Functional heartburn	1.7 (0.9–2.0)	0.6 (0.1–2.0)	2.2 (1.0–4.2)	2.0 (1.0–3.3)	0.287
Reflux hypersensitivity	1.1 (0.4–2.3)	0.6 (0.1–2.0)	2.5 (1.2–4.5)	1.8 (0.9–3.1)	0.118
Globus	1.9 (1.0–3.3)	0.6 (0.1–2.0)	2.5 (1.2–4.5)	1.5 (0.7–2.8)	0.198
Functional dysphagia	3.0 (1.8–4.6)	3.1 (1.5–5.4)	5.7 (3.6–8.4)	5.0 (3.5–6.9)	0.079
Any gastroduodenal DGBI	12.1 (9.7–14.9)	10.6 (7.6–14.2)	12.6 (9.5–16.3)	14.6 (12.0–17.6)	0.272
Functional dyspepsia	8.3 (6.3–10.8)	7.5 (5.0–10.7)	10.4 (7.6–13.8)	9.8 (7.6–12.3)	0.425
PDS	7.4 (5.5–9.7)	7.2 (4.8–10.4)	9.4 (6.7–12.)	8.4 (6.4–10.8)	0.607
EPS	3.1 (1.9–4.8)	1.1 (0.3–2.8)	4.5 (2.7–7.0)	3.8 (2.5–5.5)	0.053
Belching disorder	0.8 (0.3–1.8)	1.1 (0.3–2.8)	1.7 (0.7–3.5)	1.1 (0.4–2.2)	0.564
Rumination syndrome	3.1 (1.9–4.8)	3.6 (1.9–6.1)	2.7 (1.4–4.8)	5.1 (3.6–7.1)	0.152
Chronic nausea/vomiting	1.1 (0.4–2.3)	0.3 (0.0–1.5)	1.2 (0.4–2.9)	1.2 (0.5–2.4)	0.488
Cyclic vomiting syndrome	2.2 (1.2–3.7)	1.7 (0.6–3.6)	1.2 (0.4–2.9)	1.8 (0.9–3.1)	0.719
Cannabinoid hyperemesis	0.0 (0.0–0.6)	0.0 (0.0–1.0)	0.0 (0.0–0.9)	0.0 (0.0–0.6)	> 0.999
Any bowel DGBI	36.2 (32.4–40.0)	35.0 (30.1–40.2)	36.6 (31.9–41.5)	39.7 (35.9–43.5)	0.420
Rome IV IBS	4.2 (2.8–6.1)	3.9 (2.1–6.4)	5.7 (3.6–8.4)	5.9 (4.2–8.0)	0.362
IBS-C	1.1 (0.4–2.3)	1.7 (0.6–3.6)	0.5 (0.1–1.8)	2.3 (1.3–3.7)	0.099
IBS-D	2.0 (1.1–3.5)	1.9 (0.8–4.0)	2.7 (1.4–4.8)	2.1 (1.2–3.5)	0.868
IBS-M	0.9 (0.3–2.0)	0.3 (0.0–1.5)	2.0 (0.9–3.9)	1.5 (0.7–2.8)	0.140
IBS-U	0.2 (0.0–0.9)	0.0 (00.-1.0)	0.5 (0.1–1.8)	0.0 (0.0–0.6)	0.180
Functional constipation	12.6 (10.1–15.4)	15.8 (12.2–20.0)	14.1 (10.9–1.9)	13.9 (11.3–16.7)	0.558
Opioid-induced constipation	1.4 (0.6–2.7)	1.7 (0.6–3.6)	0.7 (0.2–2.2)	0.8 (0.2–1.8)	0.423
Functional diarrhea	3.3 (2.1–5.0)	2.8 (1.3–5.0)	2.2 (0.1–4.2)	2.9 (1.7–4.4)	0.792
Functional abdominal bloating/distention	3.5 (2.2–5.2)	3.1 (1.5–5.4)	5.2 (3.2-.8)	5.0 (3.5–6.9)	0.260
Unspecified functional bowel disorder	11.0 (8.7–13.7)	8.1 (5.5–11.4)	8.7 (6.1–11.8)	11.3 (9.0–14.0)	0.240
Centrally mediated abdominal pain syndrome	0.0 (0.0–0.6)	0.0 (0.0–1.0)	0.0 (0.0–0.9)	0.2 (0.0–0.8)	0.549
Functional biliary pain	0.0 (0.0–0.6)	0.3 (0.0–1.5)	0.2 (0.0–1.4)	0.0 (0.0–0.6)	0.331
Any anorectal DGBI	10.2 (8.0–12.8)	8.6 (5.9–12.0)	9.9 (7.2–13.2)	11.9 (9.5–14.6)	0.393
Fecal incontinence	1.4 (0.6–2.7)*	0.0 (0.0–1.0)*	0.2 (0.0–1.4)*	0.9 (0.3–2.0)*	0.050
Levator ani syndrome	1.6 (0.8–2.9)	1.1 (0.3–2.8)	1.7 (0.7–3.5)	1.8 (0.9–3.1)	0.854
Proctalgia fugax	7.5 (5.6–9.9)	7.5 (5.0–10.7)	7.9 (5.5–11.0)	9.4 (7.2–11.8)	0.614
Number of DGBI/person	0.71 (0.63–0.79)	0.64 (0.54–0.75)	0.79 (0.67–0.91)	0.84 (0.75–0.93)	0.063
Number of GI anatomic regions with DGBI, mean (95% CI)	0.65 (0.58–0.72)	0.59 (0.50–0.68)	0.69 (0.60–0.79)	0.76 (0.69–084)	0.057
Number of GI anatomic regions with DGBI					
One GI anatomic regions with DGBI	26.4 (23.0–30.0)	25.3 (20.9–30.1)	28.7 (24.3–33.4)	27.5 (24.1–31.0)	0.724
Two GI anatomic regions with DGBI	11.8 (9.4–14.6)	10.0 (7.1–13.6)	9.7 (7.0–13.0)	12.8 (10.4–15.6)	0.324
Three GI anatomic regions with DGBI	4.1 (2.7–5.9)	3.6 (1.9–6.1)	4.0 (2.3–6.4)	5.1 (3.6–7.1)	0.638
Four GI anatomic regions with DGBI	0.8 (0.3–1.8)	0.8 (0.2–2.4)	2.5 (1.2–4.5)	2.0 (1.0–3.3)	-

Northwest includes: Aosta Valley, Piedmont, Lombardy and Liguria

Northeast includes: Friuli-Venezia Giulia, Veneto, Trentino-Alto Adige, and Emilia-Romagna

Center includes: Tuscany, Marche, Umbria, and Lazio

South and Islands include: Abruzzo, Molise, Campania, Apulia, Basilicata, Calabria, Sicily and Sardinia

Abbreviations: DGBI, Disorder of the brain-gut interaction; EPS, Epigastric pain syndrome; IBS-C, Constipation predominant irritable bowel syndrome; IBS-D, Diarrhea predominant irritable bowel syndrome; IBS-M, Mixed irritable bowel syndrome; IBS-U, Unspecified irritable bowel syndrome;; PDS, Postprandial distress syndrome

^*^Comparisons within Italian population, *P* value < 0.05; ** Comparisons within Italian population, *P* value < 0.01; *** Comparisons within Italian population, *P* value < 0.001

### Prevalence of specific DGBI

The prevalence of FD was 9.1% (Fig. 2B). The overall prevalence of IBS in Italy was 5.0%, with subtype distribution as follows: IBS-C 1.5%, IBS-D 2.2%, IBS-M 1.2%, and IBS-U 0.1%. The prevalence of FC was 13.9%, and FDr was 2.9% (Table 1). When examining regional prevalence patterns across Italy, notable differences emerged among the main DGBI. FD was most prevalent in the Central region (10.4%), followed by the South and Islands (9.8%), Northwest (8.3%), and Northeast (7.5%). IBS was most prevalent in the South and Islands (5.9%), followed closely by the Central region (5.7%), with lower rates observed in the Northwest (4.2%) and Northeast (3.9%). The prevalence of FC was relatively high and consistent across regions, with the highest rates reported in the Northeast (15.8%) and Central region (14.1%), followed by the South and Islands (13.9%), and the Northwest (12.6%). Lastly, FDr showed the highest prevalence in the Northwest (3.3%), followed by the South and Islands (2.9%), the Northeast (2.8%), and the Central region (2.2%) (Table 2).

### Comparison of DGBI prevalence between Italy and other European countries

The prevalence of at least one DGBI was significantly higher in Italy than the rest of Europe (44.2% vs. 39.5%, *p* < 0.001) (Supplementary Table 1). This difference was observed consistently across most gastrointestinal anatomical regions. Esophageal DGBI were more common in Italy than in the rest of Europe (8.4% vs. 6.0%, *p* < 0.001). Similarly, gastroduodenal DGBI were significantly more frequent in Italy (12.7% vs. 9.9%, *p* < 0.001), with FD and both of its subtypes, postprandial distress syndrome (PDS) and epigastric pain syndrome (EPS), showing higher prevalence rates in Italy. Bowel DGBI represented the most prevalent group in both populations, with higher rates in Italy compared with Europe (37.2% vs. 32.9%, *p* < 0.001). Rome IV-defined IBS was more common in Italy, largely driven by a higher prevalence of IBS-D, while no significant differences were observed for IBS-C, IBS-M, or IBS-U. On the contrary, functional diarrhea was more frequent in the European population. Anorectal DGBI were significantly more prevalent in Italy than in the rest of Europe (10.4% vs. 7.3%, *p* < 0.001), mainly due to a higher prevalence of proctalgia fugax. In contrast, fecal incontinence was slightly more common in the European cohort, while levator ani syndrome showed no significant difference. Finally, Italians exhibited a greater overall disease burden, with a higher mean number of DGBI per person (0.76 vs. 0.61, *p* < 0.001) and a higher mean number of affected gastrointestinal anatomical regions (0.69 vs. 0.56, *p* < 0.001). While the proportion of individuals with involvement of a single anatomical region was comparable between groups, in comparison to the European cohort, Italians showed significantly higher proportions of individuals with overlapping DGBI, with higher proportions of individuals presenting DGBI affecting two (11.4% vs. 8.9%), three (4.3% vs. 2.5%), or four gastrointestinal anatomical regions (1.5% vs. 0.9%).

### Risk factors associated with DGBI within Italy

**Fig. 3:**
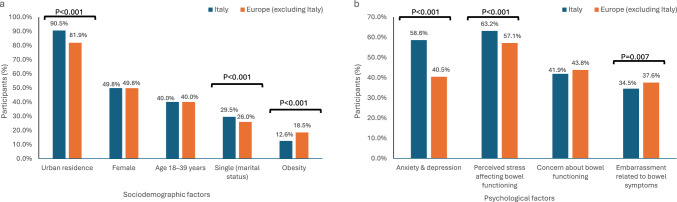
**a** Sociodemographic and **b** psychological characteristics according to geographical area (Italy compared with other European regions)

In the univariate analysis (Supplementary Table 2 and Fig. 3), several sociodemographic, psychological, and lifestyle factors were consistently associated with DGBI in Italy. Across both samples, being female (Italy: OR = 2.19; Europe: OR = 1.79), younger age (OR = 0.98 per year in both), presence of anxiety and depression (Italy: OR = 3.26; Europe: OR = 3.12), and higher somatic symptom burden (Italy: OR = 1.23; Europe: OR = 1.24) were strongly associated with DGBI (*p* < 0.001 for all), suggesting a shared underlying psychosocial profile. In addition, underweight status was more strongly associated with DGBI in Italy (OR = 1.96, *p* = 0.006) compared to Europe (OR = 1.61, *p* < 0.001), while obesity showed inverse trends between the two populations. Rural living was not significantly associated with DGBI in Italy overall, but was protective in Europe (OR = 0.86, *p* < 0.001). Notably, living in a very small non-urban countryside area (less than 2,500 inhabitants) was significantly less common among patients with DGBI in Italy (OR = 0.35, *p* = 0.037), suggesting a more nuanced role of environment at the local level. Patterns of healthcare use and impact on functioning were similar across regions. Patients with DGBI in both Italy and Europe reported more frequent doctor visits, higher use of gastrointestinal-related medications, and greater impairment in work productivity and daily activity.

Dietary differences between European and Italian cohorts were also noted. In both samples, DGBI patients consumed fewer fruits, vegetables and legumes, and were more likely to avoid specific foods. In Italy, significant avoidances included vegetables and legumes (OR = 1.75, *p* = 0.005) and wheat-based products, such as bread and pasta (OR = 1.92, *p* = 0.003).

Beyond their association with demographic and psychological factors, DGBIs in Italy were accompanied by a markedly higher clinical burden. Compared to non-DGBI subjects, those with DGBI reported nearly two-fold higher rates of concern or embarrassment about bowel function (56.7% vs. 30.1%,45.1% vs 26.2%, respectively) and made significantly greater use of symptom-targeted medications, particularly laxatives (11.1% vs. 3.9%), antidiarrheals (7.1% vs. 3.3%), antiemetics (7.0% vs. 2.0%), antacids (35.0% vs. 16.2%), and agents for gas or bloating (13.3% vs. 6.3%) (all *p* < 0.001).

These univariate patterns were further explored in multivariate logistic regression models (Table 3), which adjusted for potential confounders and allowed for a clearer identification of independent associations. Across Italy, female sex (Italy: OR = 1.69; Europe: OR = 1.38; *p* < 0.001 for both), younger age (Italy: OR = 1.35; Europe: OR = 1.12), the presence of anxiety and depression (Italy: OR = 2.03; Europe: OR = 1.84), and higher somatic symptom burden (OR = 1.18 in both populations; *p* < 0.001) remained robust and statistically significant associated with DGBI. Interestingly, the environmental context showed region-specific effects. In the Italian model, residing in a very small, non-urban countryside location was independently associated with a lower likelihood of DGBI (OR = 0.27, *p* = 0.024). In contrast, rural residence as a broader category was not significant in the Italian model but was inversely associated with DGBI in the European model (OR = 0.90, *p* = 0.024). Additionally, living in Southern European countries, including Italy, was associated with DGBI in the European-level analysis (OR = 1.17, *p* < 0.001). For FD, anxiety and/or depression more than doubled the odds of FD in Italy (OR 2.18) and Europe (OR 2.07), while increasing somatic symptom burden was strongly associated with FD in both models (Italy OR 1.27; Europe OR 1.22). In Europe, but not in Italy, younger age (OR 1.22), underweight status (OR 1.67), and residence in southern European countries (OR 1.19) were additional independent correlates of FD. Similarly, for IBS, psychosocial factors exerted the strongest influence across populations. Anxiety and/or depression were associated with a more than three-fold increase in IBS odds in Italy (OR 3.26) and a 2.6-fold increase in Europe (OR 2.59), while higher somatic symptom burden was consistently associated with IBS in both settings (OR 1.24). In the European model only, female sex (OR 1.28), younger age (OR 1.38), and residence in southern European countries (OR 1.17) remained independently associated with IBS.

**Table 3:** Multivariate analysis assessing factors associated with any DGBI, functional dyspepsia and IBS occurrence in Italy and Europe

	Multivariate–Italy		Multivariate–Europe^4^	
	OR (95% CI)	*P* value	OR (95% CI)	*P* value
*Any DGBI*				
Gender (female)	1.69 (1.39–2.07)	0.001	1.38 (1.30–1.48)	< 0.001
Age (18–39 years)	1.35 (1.10–1.64)	0.004	1.12 (1.04–1.19)	0.001
Presence of anxiety and depression (mild, moderate, severe)^1^	2.03 (1.64–2.51)	< 0.001	1.84 (1.72–1.97)	< 0.001
Somatic symptoms scale^2^	1.18 (1.14–1.22)	< 0.001	1.18 (1.17–1.20)	< 0.001
Rural living area	N.A	N.A	0.90 (0.83–0.99)	0.024
Southern European countries^3^	N.A	N.A	1.17 (1.08–1.26)	< 0.001
Living in a place in the countryside that is not part of any city, town or village	0.27 (0.09–0.85)	0.024	N.A	N.A
*Functional Dyspepsia*				
Age (18–39 years)	N.A	N.A	1.22 (1.08–1.37)	0.001
Presence of any degree of anxiety and depression (mild, moderate, or severe) ^1^	2.18 (1.42–3.34)	< 0.001	2.07 (1.80–2.37)	< 0.001
Somatic symptoms scale^2^	1.27 (1.21–1.32)	< 0.001	1.22 (1.19–1.23)	< 0.001
Underweight (BMI < 18.5 kg/m^2^)	N.A	N.A	1.67 (1.31–2.13)	< 0.001
Southern European countries^3^	N.A	N.A	1.19 (1.03–1.36)	0.015
*IBS*				
Gender (female)	N.A	N.A	1.28 (1.09–1.49)	0.003
Age (18–39 years)	N.A	N.A	1.38 (1.18–1.61)	< 0.001
Presence of anxiety and depression (mild, moderate, or severe)^1^	3.26 (1.68–6.29)	< 0.001	2.59 (2.14–3.15)	< 0.001
Somatic symptoms scale^2^	1.24 (1.17–1.31)	< 0.001	1.24 (1.22–1.26)	< 0.001
Southern European countries^3^	N.A	N.A	1.17 (1.01–1.38)	0.048

IBS, Irritable bowel syndrome; N.A., Not applicable**.** ^1^Anxiety and depression were evaluated by the PHQ-4 questionnaire, while 0–2 score relates to non-psychological distress, 3–5 score = mild psychological distress, 6–8 score = moderate psychological distress, and 9–12 score = severe psychological distress. ^2^Somatic symptom scale score was evaluated by the PHQ-12 questionnaire, without 3 gastrointestinal items and a menstrual symptom item. ^3^Southern European countries include Italy and Spain. ^4^European population includes the following countries: Italy, Spain, Sweden, Romania, Belgium, France, Germany, the Netherlands, Poland, and the United Kingdom

## Discussion

The RFGES provides a uniquely large and multi-national perspective on DGBI, encompassing over 54,000 adults across 26 countries. In Italy, specifically, a representative sample of 2,063 adults was assessed, with roughly 44% reporting one or more DGBI. Italy’s DGBI rates broadly reflect the range seen in other Western countries although with significant variation both within the country and with the rest of Europe [6].

A key finding of the present study is that the burden of DGBI varies substantially across Italian regions. We observed the highest overall DGBI prevalence in the South and Islands, with progressively lower rates in Central, Northwest, and Northeast Italy. In particular, the prevalence of IBS and FD was greater in southern and Central Italy, while FC and FDr only showed modest variations. However, while in the univariate analysis, we found a significant association between at least one DGBI and living in Italian Southern and Islands regions, this association was not confirmed in the multivariate analysis.

Psychological distress emerged as a strong shared associated factor for DGBI across Italy and Europe. In our study, the presence of anxiety and depression doubled the odds of having a DGBI (Italy: OR = 2.03; Europe: OR = 1.84). This further highlights the biopsychosocial nature of DGBI: brain–gut axis perturbations are fundamental, and psychological factors influence disease severity. [13] Notably, a recent Italian survey from the Italian Statistics Institute (ISTAT) found higher rates of impaired mental health measured by the questionnaire short form-36 (F-36) in southern regions compared with northern areas. [14] It could be hypothesized that the lower overall socioeconomic status found in the southern region, among other factors, may impact the psychological well-being of individuals, which, in turn, may reflect on gut health with higher DGBI rates. These findings contrast partially with pediatric studies, indeed a large survey of Italian schoolchildren found no significant North–South difference in overall DGBI prevalence, likely showing that psychosocial causes may have a cumulative effect in later phases of life [15].

The comparison of Italian data with other European countries reveals that Italy bears a significantly higher burden of both overall DGBI and specific DGBI than the rest of Europe. Within Italy, a similar South–North gradient was observed, with the southern regions showing higher DGBI rates than the northern regions. Notably, adherence to the traditional Mediterranean diet, which is more common in Italy than the rest of Europe (and more common in southern Italy than northern Italy) might be expected to lower DGBI, [16] but this diet also includes FODMAP-rich foods like legumes, wheat, and dairy. Thus, while a Mediterranean lifestyle is often considered beneficial for both general health and DGBI, it may trigger IBS symptoms in sensitive individuals [17]. This paradox mirrors what has been reported in previously published data, where data on the association between the Mediterranean diet and DGBI are inconsistent. In fact, a recently published systematic review and network metanalysis on the efficacy of different diets in IBS showed that the Mediterranean diet was associated with lower rates of IBS general symptoms [16]. Specific data on the Mediterranean diet were retrieved from two randomized controlled studies[18, 19] on 85 patients, showing an OR of 0.27 (95% confidence interval (CI) 0.11–0.67). However, a randomized control trial on 106 IBS patients assessing the adherence to Mediterranean diet compared to a healthy population showed that a standard Mediterranean diet was not associated with IBS symptom severity, although certain foods were associated with increased IBS symptoms, suggesting that standard Mediterranean diet may not be suitable for all patients with IBS and likely needs to be personalized in those with increased symptoms [17]. Future work should clarify how regional culture, diet, and environment interact to produce the observed gradient in DGBI prevalence.

Unmeasured confounders like socioeconomic status may have influenced the results also. Previous data on rates of DGBI in Italy showed higher IBS rates in cities compared to the countryside using Rome II diagnostic criteria (9.9% vs 4.4%) [20]. However, this study was limited to only two specific rural communities in Italy, while our study gives insights into a more geographically representative national population. On the other hand, as for self-reported FD, we found slightly higher rates of FD when compared to a previous endoscopy-based study using Rome III criteria, reporting a prevalence of 11% in the Italian general population [5]. Moreover, it was not possible to confirm the association of FD with employment and smoking as previously reported.

Our study has several limitations. The RFGES used internet-based questionnaires in Italy, which might under-represent the elderly population or those without internet access. Self-report of diet and symptoms can introduce recall bias. Nevertheless, the use of validated Rome IV modules, the large and representative sample, and the consistency with established epidemiology reinforce the findings and are important strengths. An additional limitation is the lack of procedures such as endoscopy or manometry, a small part of patients classified as DGBI may have underlying “organic” diseases. However, since the methods of data acquisition were the same both within Italy and within Europe, this does not likely modify the differences observed between populations.

In conclusion, this comprehensive Italian analysis of the RFGES highlights a high national burden of DGBI, with distinct regional patterns and strong links to psychosocial and dietary factors. DGBIs afflict men and women in Italy far more often than previously reported, especially in the South, and impose significant health and economic burdens. Moreover, the differences between southern European and northern, western and eastern Europe are likely due to different dietary styles, socioeconomic conditions, and healthcare access, rather than genetic factors. Future studies should investigate the causal mechanisms for the north–south gradient in Italy and Europe, such as regional differences in stress exposure, medical culture, or unmeasured environmental factors. Longitudinal studies could clarify the roles of dietary change and acculturation, and whether Mediterranean lifestyle promotion might reduce DGBI incidence over time.

## Supplementary Information

Below is the link to the electronic supplementary material.

Supplementary file1 (DOCX 89 KB)

## Data Availability

Data are available upon reasonable request, subject to approval by the Rome Foundation.
