# Supplementary material for: Burden, features and factor associated with disorders of gut–brain axis in Italy: results from the Rome foundation global epidemiology study
Source: Intern Emerg Med. 2026 Apr 30;21(5):1655–67. doi: 10.1007/s11739-026-04350-w (PMC13452765; doi:10.1007/s11739-026-04350-w)
Supplement: Supplementary file 1 [file 11739_2026_4350_MOESM1_ESM.docx]

**Supplementary Table 1. Prevalence rate (%, 95% CI) for 22 DGBI among the Italian and European population**

|  | Itatly (n=2,063) | Europe (excluding Italy) (n=18,357) | P-value |
| --- | --- | --- | --- |
| Any DGBI | 44.2 (42.1–46.4) | 39.5 (38.8–40.2) | <0.001 |
| Any esophageal DGBI | 8.4 (7.3–9.7) | 6.0 (5.6–6.3) | <0.001 |
| Functional chest pain | 1.6 (1.2–2.3) | 1.7 (1.5–1.9) | 0.964 |
| Functional heartburn | 2.0 (1.5–2.7) | 1.1 (1.0–1.3) | <0.001 |
| Reflux hypersensitivity | 1.7 (1.2–2.4) | 0.9 (0.8–1.0) | <0.001 |
| Globus | 1.7 (1.2–2.4) | 0.8 (0.7–1.0) | <0.001 |
| Functional dysphagia | 4.8 (4.0–5.8) | 3.5 (3.2–3.7) | 0.002 |
| Any gastroduodenal DGBI | 12.7 (11.4–14.3) | 9.9 (9.5–10.4) | <0.001 |
| Functional dyspepsia | 10.0 (8.8–11.4) | 7.9 (7.5–8.3) | <0.001 |
| PDS | 8.8 (7.6–10.1) | 6.5 (6.1–6.8) | <0.001 |
| EPS | 4.0 (3.2–4.9) | 2.9 (2.7–3.2) | 0.008 |
| Belching disorder | 1.4 (0.9–2.0) | 0.9 (0.8–1.1) | 0.048 |
| Rumination syndrome | 4.1 (3.3–5.1) | 2.6 (2.4–2.9) | <0.001 |
| Chronic nausea/vomiting | 1.1 (0.7–1.7) | 1.1 (1.0–1.3) | 0.889 |
| Cyclic vomiting syndrome | 2.2 (1.7–3.0) | 1.1 (1.0–1.3) | <0.001 |
| Cannabinoid hyperemesis | 0.0 (0.0–0.2) | 0.0 (0.0–0.1) | 0.612 |
| Any bowel DGBI | 37.2 (35.1–39.3) | 32.9 (32.2–33.6) | <0.001 |
| Rome IV IBS | 5.9 (5.0–7.0) | 4.6 (4.3–5.0) | 0.010 |
| IBS-C | 1.5 (1.0–2.1) | 1.2 (1.0–1.4) | 0.305 |
| IBS-D | 2.2 (1.6–2.9) | 1.2 (1.0–1.3) | <0.001 |
| IBS-M | 1.2 (0.8–1.8) | 1.3 (1.1–1.5) | 0.762 |
| IBS-U | 0.1 (0.0–0.4) | 0.2 (0.2–0.3) | 0.621 |
| Functional constipation | 14.9 (13.5–16.5) | 12.0 (11.6–12.5) | <0.001 |
| Opioid-induced constipation | 1.3 (0.9–1.8) | 1.5 (1.4–1.7) | 0.321 |
| Functional diarrhea | 3.6 (2.9–4.5) | 5.0 (4.7–5.3) | 0.005 |
| Functional abdominal bloating/distention | 4.6 (3.7–5.5) | 4.1 (3.8–4.4) | 0.332 |
| Unspecified functional bowel disorder | 10.8 (9.5–12.2) | 9.4 (8.9–9.8) | 0.039 |
| Centrally mediated abdominal pain syndrome | 0.0 (0.0–0.3) | 0.0 (0.0–0.1) | 0.413 |
| Functional biliary pain | 0.1 (0.0–0.4) | 0.1 (0.1–0.2) | >0.999 |
| Any anorectal DGBI | 10.4 (9.2–11.8) | 7.3 (6.9–7.7) | <0.001 |
| Fecal incontinence | 1.3 (0.9–1.8) | 1.9 (1.7–2.1) | 0.037 |
| Levator ani syndrome | 1.8 (1.3–2.5) | 1.3 (1.1–1.5) | 0.067 |
| Proctalgia fugax | 9.2 (8.0–10.5) | 5.9 (5.5–6.2) | <0.001 |
| Number of DGBI/person | 0.76 (0.71–0.81) | 0.61 (0.59–0.62) | <0.001 |
| Number of GI anatomic regions with DGBI, mean (95% CI) | 0.69 (0.65–0.73) | 0.56 (0.55–0.57) | <0.001 |
| Number of GI anatomic regions with DGBI |  |  |  |
| One GI anatomic regions with DGBI | 27.0 (25.1–29.0) | 27.2 (26.6–27.9) | 0.814 |
| Two GI anatomic regions with DGBI | 11.4 (10.1–12.8) | 8.9 (8.5–9.3) | <0.001 |
| Three GI anatomic regions with DGBI | 4.3 (3.5–5.3) | 2.5 (2.3–2.8) | <0.001 |
| Four GI anatomic regions with DGBI | 1.5 (1.1–2.1) | 0.9 (0.8–1.0) | 0.005 |

Eropean countries include: Belgium, France, Germany, Holland, Poland, Romanian, Sweden, United Kingdom, and Spain

Abbreviations: DGBI, Disorder of the brain-gut interaction; EPS, Epigastric pain syndrome; IBS-C, Constipation predominant irritable bowel syndrome; IBS-D, Diarrhea predominant irritable bowel syndrome; IBS-M, Mixed irritable bowel syndrome; IBS-U, Unspecified irritable bowel syndrome;; PDS, Postprandial distress syndrome

**Supplemental Table 2. Risk factors for DGBI among Italian and European population**

|  | Italian population without DGBI (n=1151) | Italian population with at least 1 DGBI (n=912) | Unadjusted OR (95% CI) | P-value | European population without DGBI (n=12250) | European population with at least 1 DGBI (n=8170) | Unadjusted OR (95% CI) | P-value |
| --- | --- | --- | --- | --- | --- | --- | --- | --- |
| **Sociodemographic information** |  |  |  |  |  |  |  |  |
| Female, % (95% CI) | 41.3 (38.4-44.2) | 60.6 (57.4-63.8) | 2.19 (1.83-2.62) | <0.001 | 44.0 (43.1-44.9) | 58.5 (57.4-59.6) | 1.79 (1.69-1.89) | <0.001 |
| Age (years), mean (95% CI) | 47.7 (46.7-48.6) | 42.9 (41.9-43.9) | 0.98 (0.97-0.98) | <0.001 | 47.3 (47.01-47.6) | 43.8 (43.4-44.1) | 0.98 (0.98-0.98) | <0.001 |
| Age groups, % (95% CI) | | | | |  |  |  |  |
| - 18-39 years | 34.8 (32.0-37.6) | 46.7 (43.4-50.0) | 1.65 (1.38-1.97) | <0.001 | 37.0 (36.1-37.8) | 44.6 (43.5-45.7) | 1.37 (1.29-1.45) | <0.001 |
| - 40-64 years | 40.8 (38.0-43.7) | 39.3 (36.1-42.5) | 0.94 (0.78-1.12) | 0.467 | 41.9 (41.0-2.7) | 41.5 (40.4-42.6) | 0.98 (0.93-1.04) | 0.612 |
| - 65+ years | 24.4 (22.0-27.0) | 14.0 (11.8-16.5) | 0.51 (0.40-0.64) | <0.001 | 21.2 (20.5-21.9) | 13.9 (13.2-14.7) | 0.60 (0.55-0.65) | <0.001 |
| Education (years), mean (95% CI) | 14.7 (14.4-14.9) | 14.9 (14.-15.2) | 1.02 (0.99-1.04) | 0.129 | 13.3 (13.2-13.4) | 13.3 (13.2-13.) | 1.00 (0.99-1.00) | 0.163 |
| BMI (kg/m^2^), mean (95% CI) | 25.2 (24.9-25.4) | 24.6 (24.3-24.9) | 0.97 (0.95-0.99) | 0.004 | 25.8 (25.7-25.9) | 25.8 (257-25.9) | 1.00 (0.99-1.00) | 0.052 |
| BMI levels^1^, % (95% CI) |  |  |  |  |  |  |  |  |
| - Underweight | 2.7 (1.8-3.9) | 5.2 (3.8-6.9) | 1.96 (1.22-3.15) | 0.006 | 2.8 (2.5-3.2) | 4.5 (4.0-5.0) | 1.61 (1.37-1.88) | <0.001 |
| - Normal weight | 50.6 (47.5-53.6) | 54.1 (50.8-57.5) | 1.16 (0.96-1.38) | 0.116 | 45.5 (44.5-46.4) | 45.9 (44.8-47.0) | 1.02 (0.96-1.08) | 0.576 |
| - Overweight | 32.9 (30.1-35.8) | 29.7 (26.7-32.9) | 0.86 (0.71-1.05) | 0.139 | 34.6 (33.8-35.6) | 30.5 (29.5-31.6) | 0.83 (0.77-0.88) | <0.001 |
| - Obesity | 13.9 (11.8-16.1) | 10.9 (8.9-13.2) | 0.76 (0.58-1.01) | 0.055 | 17.1 (16.4-17.8) | 19.1 (18.2-20.0) | 1.15 (1.06-1.24) | <0.001 |
| Family status, % (95% CI) | | | | |  |  |  |  |
| - Single | 29.1 (26.5-31.8) | 30.0 (27.1-33.1) | 1.05 (0.87-1.27) | 0.642 | 26.0 (25.3-26.8) | 26.8 (25.8-27.8) | 1.04 (0.97-1.11) | 0.237 |
| - Married | 47.8 (44.9-50.7) | 45.6 (42.3-48.9) | 0.92 (0.77-1.09) | 0.327 | 47.8 (47.0-48.7) | 44.8 (43.8-45.9) | 0.89 (0.84-0.94) | <0.001 |
| - Divorced | 7.0 (5.5-8.6) | 6.1 (4.7-7.9) | 0.88 (0.62-1.25) | 0.462 | 7.6 (7.1-8.0) | 8.1 (7.5-8.7) | 1.08 (0.97-1.19) | 0.165 |
| - Widowed | 2.3 (1.6-3.4) | 2.9 (1.9-4.1) | 1.22 (0.71-2.11) | 0.472 | 3.2 (2.9-3.5) | 2.4 (2.1-2.7) | 0.74 (0.62-0.88) | <0.001 |
| - Co-habiting | 13.8 (11.9-15.9) | 15.4 (13.1-17.9) | 1.13 (0.88-1.45) | 0.325 | 15.4 (14.7-16.0) | 17.9 (17.1-18.8) | 1.20 (1.11-1.29) | <0.001 |
| Rural living area, % (95% CI) | 10.3 (8.6-12.2) | 8.3 (6.6-10.3) | 0.78 (0.58-1.06) | 0.123 | 18.1 (17.4-18.8) | 16.0 (15.2-16.8) | 0.86 (0.80-0.93) | <0.001 |
| Size of local community^2^, % (95% CI) | | | | |  |  |  |  |
| - City | 48.7 (45.8-51.7) | 49.0 (45.7-52.3) | 1.01 (0.85-1.20) | 0.902 | 46.5 (45.6-47.4) | 49.2 (48.2-50.3) | 1.12 (1.06-1.18) | <0.001 |
| - Town | 40.9 (38.1-43.8) | 42.7 (39.4-45.9) | 1.07 (0.90-1.28) | 0.428 | 35.5 (34.6-36.6) | 34.8 (33.7-35.8) | 0.97 (0.91-1.03) | 0.316 |
| - Village | 8.8 (7.2-10.6) | 7.8 (6.1-9.7) | 0.88 (0.64-1.21) | 0.420 | 14.4 (13.8-15.1) | 13.1 (12.4-13.9) | 0.89 (0.82-0.97) | 0.008 |
| - Place in the countryside that is not part of any city, town or village | 1.6 (0.9-2.5) | 0.5 (0.2-1.3) | 0.35 (0.13-0.94) | 0.037 | 3.7 (3.3-4.0) | 2.9 (2.5-3.3) | 0.78 (0.66-0.92) | 0.003 |
| Italian geographical region, % (95% CI) |  |  |  |  |  |  |  |  |
| - North-West | 31.5 (28.8-34.2) | 30.0 (27.1-33.1) | 0.94 (0.77-1.13) | 0.492 | N.A. | N.A. | N.A. | N.A. |
| - North-East | 18.9 (16.6-21.2) | 15.7 (13.4-18.2) | 0.80 (0.63-1.01) | 0.060 | N.A. | N.A. | N.A. | N.A. |
| - Center | 19.4 (17.1-21.8) | 19.8 (17.3-22.6) | 1.03 (0.83-1.28) | 0.788 | N.A. | N.A. | N.A. | N.A. |
| - South and Islands | 30.3 (27.7-33.1) | 34.4 (31.3-37.6) | 1.21 (1.00-1.45) | 0.047 | N.A. | N.A. | N.A. | N.A. |
| **General assessments** |  |  |  |  | N.A. | N.A. | N.A. | N.A. |
| Global physical health (PROMIS Global-10), mean (95% CI) | 15.2 (15.1-15.4) | 13.7 (13.6-13.9) | 0.79 (0.77-0.82) | <0.001 | 15.2 (15.1-15.2) | 13.4 (13.3-13.5) | 0.78 (0.77-0.79) | <0.001 |
| Global mental health (PROMIS Global-10), mean (95% CI) | 13.8 (13.6-14.0) | 12.3 (12.1-12.6) | 0.85 (0.83-0.88) | <0.001 | 14.4 (14.3-14.5) | 12.7 (12.6-12.8) | 0.85 (0.84-0.85) | <0.001 |
| Somatic symptoms scale score^3^, mean (95% CI) | 4.1 (3.9-4.3) | 6.3 (6.1-6.5) | 1.23 (1.19-1.27) | <0.001 | 5.1 (5.0-5.2) | 8.8 (8.6-8.8) | 1.24 (1.23-1.25) | <0.001 |
| Fibromyalgia syndrome^4^, % (95% CI) | 1.4 (1.3-1.6) | 2.3 (1.4-3.5) | 1.67 (0.87-3.22) | 0.125 | 1.4 (1.2-1.6) | 3.1 (2.7-3.5) | 2.27 (1.83-2.71) | <0.001 |
| **Psychological variables** |  |  |  |  |  |  |  |  |
| Presence of anxiety and depression^5^, % (95% CI) | 46.5 (43.6-49.4) | 73.9 (70.9-76.7) | 3.26 (2.70-3.93) | <0.001 | 31.3 (30.5-32.1) | 58.5 (57.7-59.8) | 3.12 (2.95-3.31) | <0.001 |
| Anxiety and depression score^5^, mean (95% CI) | 2.8 (2.6-2.9) | 4.5 (4.3-4.7) | 1.25 (1.21-1.29) | <0.001 | 1.94 (1.89-1.98) | 3.76 (3.69-3.83) | 1.25 (1.23-1.26) | <0.001 |
| Concerns and embarrassment regarding bowel functioning |  |  |  |  |  |  |  |  |
| - Concern regarding bowel functioning (somewhat/very concerned), % (95% CI) | 30.1 (27.5-32.9) | 56.7 (53.4-59.9) | 3.03 (2.53-3.63) | <0.001 | 31.4 (30.6-32.2) | 62.0 (60.9-63.0) | 3.56 (3.35-3.77) | <0.001 |
| - Embarrassment discussing bowel function with family or friends, % (95% CI) | 26.2 (23.6-28.8) | 45.1 (41.8-48.4) | 2.32 (1.92-2.78) | <0.001 | 30.7 (29.9-31.5) | 47.1 (46.0-48.2) | 2.00 (1.89-2.12) | <0.001 |
| - Stress, pressure or tension affecting bowel functioning, % (95% CI) | 49.3 (46.3-52.2) | 80.7 (78.0-83.2) | 4.31 (3.52-5.27) | <0.001 | 46.1 (45.2-47.0) | 75.2 (74.2-76.1) | 3.54 (3.32-3.76) | <0.001 |
| **General and bowel related healthcare usage** |  |  |  |  |  |  |  |  |
| Doctor visits for bowel problem, % (95% CI) | 34.6 (31.8-37.4) | 53.7 (50.4-57.0) | 2.19 (1.84-2.62) | <0.001 | 26.2 (25.2-26.8) | 45.0 (44.0-46.1) | 2.33 (2.19-2.47) | <0.001 |
| Frequency of doctor visits, % (95% CI): |  |  |  |  |  |  |  |  |
| - Never | 1.9 (1.2-2.9) | 1.3 (0.7-2.3) | 0.68 (0.34-1.39) | 0.294 | 2.8 (2.6-3.2) | 1.8 (1.5-2.1) | 0.62 (0.51-0.76) | <0.001 |
| - Less than once a year | 14.8 (12.8-17.0) | 10.0 (8.1-12.1) | 0.64 (0.48-0.84) | 0.001 | 22.2 (21.5-23.0) | 15.4 (14.7-16.2) | 0.64 (0.59-0.69) | <0.001 |
| - Once a year | 10.9 (9.1-12.8) | 8.9 (7.1-10.9) | 0.80 (0.59-1.07) | 0.137 | 18.3 (17.6-19.0) | 13.7 (13.0-14.5) | 0.71 (0.66-0.77) | <0.001 |
| - A few times a year | 54.0 (51.0-56.9) | 58.1 (54.8-61.3) | 1.18 (0.99-1.41) | 0.059 | 47.9 (47.0-48.8) | 53.7 (52.7-54.8) | 1.26 (1.20-1.33) | <0.001 |
| - Once a month or more | 18.5 (16.3-20.9) | 21.7 (19.1-24.5) | 1.22 (0.98-1.52) | 0.071 | 8.8 (8.3-9.3) | 15.3 (14.5-16.1) | 1.88 (1.72-2.05) | <0.001 |
| Type of medical care that would be sought if needed, % (95% CI) | | | | |  |  |  |  |
| - Western style medicine^6^ | 34.6 (31.8-37.4) | 53.6 (50.3-56.9) | 2.18 (1.83-2.61) | <0.001 | 25.8 (25.0-26.6) | 44.8 (43.7-45.9) | 2.33 (2.20-2.47) | <0.001 |
| - Traditional / folk healer^7^ | 0.9 (0.4-1.6) | 1.8 (1.0-2.8) | 2.04 (0.92-4.51) | 0.079 | 0.8 (0.6-1.0) | 0.2 (1.7-2.4) | 2.59 (2.01-3.33) | <0.001 |
| Medications taken regularly (at least once a week), % (95% CI) |  |  |  |  |  |  |  |  |
| - Medications for constipation | 3.9 (2.9-5.2) | 11.1 (9.1-13.3) | 3.06 (2.13-4.39) | <0.001 | 2.9 (2.6-3.2) | 11.4 (10.7-12.1) | 4.30 (3.79-4.88) | <0.001 |
| - Medications for diarrhea | 3.3 (2.3-4.5) | 7.1 (5.5-9.0) | 2.25 (1.49-3.39) | <0.001 | 2.9 (2.6-3.2) | 7.0 (6.4-7.6) | 2.53 (2.21-2.90) | <0.001 |
| - Medications for nausea | 2.0 (1.3-3.0) | 7.0 (5.4-8.9) | 3.70 (2.28-6.01) | <0.001 | 2.2 (2.0-2.5) | 5.7 (5.2-6.2) | 2.62 (2.25-3.04) | <0.001 |
| - Medications for heartburn or to reduce stomach acid | 16.2 (14.1-18.4) | 35.0 (31.9-38.2) | 2.79 (2.27-3.43) | <0.001 | 13.7 (13.1-14.3) | 27.8 (26.8-28.7) | 2.42 (2.25-2.59) | <0.001 |
| - Medications for gas or bloating | 6.3 (5.0-7.9) | 13.3 (11.1-15.6) | 2.25 (1.66-3.06) | <0.001 | 4.6 (4.3-5.0) | 12.4 (11.7-13.2) | 2.92 (2.62-3.25) | <0.001 |
| - Medications for depression | 3.4 (2.4-4.6) | 9.0 (7.2-11.0) | 2.82 (1.90-4.17) | <0.001 | 6.2 (5.8-6.7) | 14.2 (13.5-15.0) | 2.50 (2.27-2.76) | <0.001 |
| **Work productivity and activity impairment^8^** |  |  |  |  |  |  |  |  |
| - % Work time missed due to health, mean (95% CI) | 2.2 (1.4-3.1) | 4.1 (3.0-5.2) | 1.00 (1.00-1.01) | 0.048 | 4.7 (4.2-5.2) | 7.3 (6.5-8.1) | 1.01 (1.01-1.01) | <0.001 |
| - % Impairment while working due to health, mean (95% CI) | 7.5 (6.0-8.9) | 15.2 (13.3-17.0) | 1.02 (1.02-1.03) | <0.001 | 7.2 (6.7-7.7) | 15.3 (14.5-16.2) | 1.02 (1.02-1.03) | <0.001 |
| - % Activity impairment due to health, mean (95% CI) | 8.1 (6.6-9.5) | 17.0 (15.0-19.0) | 1.02 (1.02-1.03) | <0.001 | 12.3 (11.8-12.7) | 24.9 (24.1-25.6) | 1.02 (1.02-1.03) | <0.001 |
| - **Diet habits** |  |  |  |  |  |  |  |  |
| - Food consumption (days/week)^9^, mean (95% CI) |  |  |  |  |  |  |  |  |
| - Milk products (milk, yogurt, or cheese) | 4.2 (4.0-4.3) | 4.3 (4.2-4.5) | 1.02 (0.98-1.06) | 0.191 | 5.0 (4.9-5.0) | 4.9 (4.8-4.9) | 0.98 (0.96-0.98) | <0.001 |
| - Meat from animals | 2.8 (2.7-2.9) | 2.9 (2.8-3.1) | 1.05 (0.99-1.11) | 0.056 | 3.9 (3.9-4.0) | 4.0 (3.9-4.0) | 1.01 (0.99-1.02) | 0.281 |
| - Fish | 1.8 (1.7-1.9) | 1.7 (1.6-1.8) | 0.91 (0.84-0.97) | 0.009 | 1.6 (1.5-1.6) | 1.6 (1.5-1.6) | 0.95 (0.93-0.97) | <0.001 |
| - Eggs | 1.6 (1.5-1.7) | 1.6 (1.5-1.7) | 0.95 (0.88-1.03) | 0.216 | 2.1 (2.1-2.2) | 2.2 (2.1-2.2) | 1.00 (0.98-1.02) | 0.966 |
| - Vegetables and legumes | 4.6 (4.5-4.8) | 4.4 (4.2-4.5) | 0.93 (0.89-0.97) | 0.002 | 4.8 (4.8-4.9) | 4.6 (4.6-4.7) | 0.95 (0.94-0.96) | <0.001 |
| - Fruits | 5.2 (5.1-5.4) | 4.8 (4.6-4.9) | 0.92 (0.88-0.95) | <0.001 | 4.7 (4.6-4.8) | 4.5 (4.4-4.5) | 0.95 (0.94-0.96) | <0.001 |
| - Bread (including pizza, pita and tortillas) | 5.2 (5.1-5.3) | 5.2 (5.0-5.3) | 0.99 (0.95-1.03) | 0.597 | 5.3 (5.2-5.4) | 5.2 (5.2-5.3) | 0.98 (0.97-0.99) | <0.001 |
| - Pasta | 4.5 (4.4-4.6) | 4.6 (4.5-4.7) | 1.02 (0.97-1.06) | 0.445 | 2.4 (2.4-2.5) | 2.6 (2.5-2.6) | 1.05 (1.03-1.07) | <0.001 |
| - Rice | 2.0 (1.9-2.1) | 1.9 (1.8-2.0) | 0.97 (0.91-1.03) | 0.266 | 1.8 (1.7-1.8) | 1.8 (1.7-1.8) | 1.02 (0.99-1.04) | 0.278 |
| - Tofu | 0.2 (0.1-0.2) | 0.2 (0.1-0.2) | 0.99 (0.88-1.11) | 0.870 | 0.2 (0.2-0.3) | 0.2 (0.2-0.3) | 1.01 (0.98-1.05) | 0.130 |
| - Food avoidance^10^, % (95% CI) | 22.3 (19.9-24.8) | 29.7 (26.7-32.8) | 1.47 (1.21-1.80) | <0.001 | N.A. | N.A. | N.A. | N.A. |
| - Specific food avoidances, % (95% CI): |  |  |  |  |  |  |  |  |
| - Milk products (milk, yogurt, or cheese) | 8.6 (7.1-10.4) | 9.5 (7.7-11.6) | 1.11 (0.82-1.50) | 0.503 | N.A. | N.A. | N.A. | N.A. |
| - Meat from animals | 3.9 (2.9-5.2) | 4.1 (2.9-5.6) | 1.04 (0.67-1.62) | 0.857 | N.A. | N.A. | N.A. | N.A. |
| - Fish and sea foods | 0.2 (0.0-0.6) | 0.6 (0.2-1.3) | 3.17 (0.61-16.39) | 0.168 | N.A. | N.A. | N.A. | N.A. |
| - Eggs | 0.7 (0.3-1.4) | 1.2 (0.6-2.2) | 1.75 (0.70-4.37) | 0.231 | N.A. | N.A. | N.A. | N.A. |
| - Vegetables and legumes | 4.1 (3.0-5.4) | 7.0 (5.4-8.8) | 1.75 (1.18-2.58) | 0.005 | N.A. | N.A. | N.A. | N.A. |
| - Fruits | 0.9 (0.4-1.6) | 1.5 (0.8-2.6) | 1.78 (0.78-4.03) | 0.165 | N.A. | N.A. | N.A. | N.A. |
| - Wheat-based grains (bread, pasta) | 3.1 (2.2-4.3) | 5.8 (4.4-7.6) | 1.92 (1.24-2.95) | 0.003 | N.A. | N.A. | N.A. | N.A. |
| - Rice | 0.2 (0.0-0.6) | 0.3 (0.1-1.0) | 1.90 (0.32-11.39) | 0.482 | N.A. | N.A. | N.A. | N.A. |
| - Coffee or tea | 1.0 (0.5-1.7) | 1.7 (0.9-2.7) | 1.74 (0.79-3.80) | 0.167 | N.A. | N.A. | N.A. | N.A. |
| - High fat foods (fried foods, sauces, fatty foods, creams) | 2.4 (1.6-3.5) | 3.9 (2.7-5.3) | 1.60 (0.97-2.66) | 0.066 | N.A. | N.A. | N.A. | N.A. |
| - Hot spices | 2.0 (1.3-3.0) | 2.2 (1.4-3.4) | 1.10 (0.60-2.02) | 0.753 | N.A. | N.A. | N.A. | N.A. |
| - Ultra processed foods^11^ | 6.3 (4.9-7.8) | 6.8 (5.3-8.7) | 1.10 (0.77-1.56) | 0.610 | N.A. | N.A. | N.A. |  |

N.A., Not available

European population includes the following countries: Italy, Spain, Sweden, Romania, Belgium, France, Germany, Netherlands, Poland and United Kingdom

^1^BMI levels: Underweight: <18.5 kg/m^2^; Normal weight: 18.5-24.9 kg/m^2^; Overweight: 25-29.9 kg/m^2^; Obesity: ≥30 kg/m^2^

^2^City - more than 50,000 inhabitants; Town - 2,500 to 50,000 inhabitants; Village or small town - less than 2,500 inhabitants.

^3^Somatic symptoms were evaluated by PHQ12 without menstrual item.

^4^Fibromyalgia syndrome was assessed by the question: "Have you ever been diagnosed by a doctor as having Fibromyalgia?"

^5^Anxiety and depression were evaluated by PHQ-4; presence of anxiety and depression was defined as any level of these disorders (i.e. mild, moderate and severe), as 0-2 score relates to none psychological distress, 3-5 score = mild psychological distress, 6-8 score = moderate psychological distress, and 9-12 score = severe psychological distress.

^6^Western style medicine includes general practitioners or family doctors, gastroenterologist, gynecologist, and surgeon.

^7^ Traditional / folk healer includes folk healer or traditional healer, homeopathic doctor, ayurvedic doctor, traditional Chinese medicine doctor, and chiropractor.

^8^Work productivity and activity impairment was assessed by WPAI Scoring:

- Percent work time missed due to health: Q2/(Q2+Q4)
- Percent impairment while working due to health: Q5/10
- Percent activity impairment due to health: Q6/10

Q2: "In the last seven days, how many hours were you away from work because of your health problems?"

Q4: "In the past seven days, how many hours did you work?"

Q5: "During the past seven days, to what extent did your health problems affect your productivity while you were working?"

Q6: "During the past seven days, to what extent did your health problems affect your ability to carry out your everyday activities other than work?"

^9^ Food items consumption was assessed using the Food Frequency Questionnaire (FFQ)

^10^ Food avoidance was assessed only in Italy via the following question: "In your experience, have you eliminated particular foods from your diet that you believe are responsible for your gastrointestinal disorders?"

^11^Ultra processed foods, include carbonated/fruit/alcoholic drinks, sausages, biscuits, packaged snacks, candies, chocolate, desserts, fast foods, and instant soups

All reported findings are based on univariate analyses.

**Specific details of administered Questionnaires and classification used in the text**

Psychological distress was evaluated using the Patient Health Questionnaire-4 (PHQ-4), a validated tool designed to measure symptoms of anxiety and depression over the past two weeks. The PHQ-4 consists of four questions scored on a four-point Likert scale, ranging from 0 ("not at all") to 3 ("nearly every day"). The total score, ranging from 0 to 12, reflects levels of anxiety, depression, and overall psychological distress, with higher scores indicating greater severity of distress. We also used a dichotomous variable to distinguish between participants with any level of psychological distress [mild (score: 3-5), moderate (score: 6-8), or severe (score: 9-12)] and those with a normal status (score: 0-2) according to validated cutoff points.

Somatic symptoms were evaluated using the Patient Health Questionnaire-12 (PHQ-12), a modified version of the PHQ-15 that excludes gastrointestinal-related items (i.e. stomach pain, constipation, loose bowels or diarrhea, and nausea, gas, or indigestion). This questionnaire consists of a list of 12 common somatic symptoms that constitute the majority of physical complaints in primary care, including one item that assessed menstrual cramps or other period-related problems. To ensure comparability between men and women, this item was excluded from the total PHQ-12 score and analyzed independently. In addition, fibromyalgia was also assessed using a single question: "Have you ever been diagnosed by a doctor as having Fibromyalgia?".

PROMIS Global-10 was used to assess both physical and mental health-related quality of life, providing validated summary scores for each domain. Higher scores reflect better self-reported health status.

The Work Productivity and Activity Impairment: General Health (WPAI: GH) questionnaire consists of six questions, with a recall period of the previous seven days. Scores for each domain range from 0% to 100%, with higher scores indicating greater impairment and lower productivity. Questions about work-related impairment (domains 1–3) were answered only by currently employed participants, while all respondents completed the question on activity impairment.

Concern and embarrassment regarding bowel functioning were assessed using the following two questions: "Are you concerned about your bowel functioning?", and "Are you embarrassed to discuss your bowel functioning with others?".

A Food Frequency Questionnaire (FFQ) was utilized to evaluate the weekly consumption frequency of various food groups, including milk products, animal-based meats, fish, eggs, vegetables and legumes, fruits, bread (including pizza, pita, and tortillas), pasta, rice, and tofu. Participants reported the number of days per week (0–7) they consumed each food group.

Food avoidance was assessed only in the Italian subsample, using a question that inquired whether participants had intentionally eliminated specific foods from their diet due to perceived gastrointestinal intolerance. Those who answered affirmatively were subsequently asked to specify the types of foods avoided. The long list of avoided food items was categorized into major food groups by a clinical dietitian (KH, RD, PhD). This information was not collected in any of the other participating countries.

The classification of residential area was based on two separate items: (1) self-reported living environment (urban vs. rural), and (2) the size of the local community, categorized as city (>50,000 inhabitants), town (2,500–50,000 inhabitants), and village/small town (<2,500 inhabitants).

Healthcare utilization was further assessed by capturing the self-reported frequency of physician visits, both in general and specifically for gastrointestinal complaints. In addition, participants were asked to report their use of medications for symptoms commonly associated with gastrointestinal disorders, including constipation, diarrhea, nausea, gastric acidity, bloating, and excessive gas. To gain deeper insight into different patterns of healthcare-seeking behaviors, participants were also asked to report the frequency with which they used Western-style medicine, which included general practitioners or family doctors, gastroenterologists, gynecologists, and surgeons; and traditional or folk medicine, which encompassed visits to folk or traditional healers, homeopathic doctors, ayurvedic doctors, traditional Chinese medicine practitioners, and chiropractors.
